# Engeletin alleviates cerebral ischemia reperfusion‐induced neuroinflammation via the HMGB1/TLR4/NF‐κB network

**DOI:** 10.1111/jcmm.17758

**Published:** 2023-05-02

**Authors:** Yangyang Xu, Jie Zhang, Fei Gao, Wenna Cheng, Ye Zhang, Chuanmei Wei, Shuping Zhang, Xinfu Gao

**Affiliations:** ^1^ School of Pharmacy Key Laboratory of Molecular Pharmacology and Drug Evaluation (Yantai University), Ministry of Education, Collaborative Innovation Center of Advanced Drug Delivery System and Biotech Drugs in Universities of Shandong, Yantai University Yantai China; ^2^ Department of Pharmacy Binzhou Medical University Hospital Binzhou China; ^3^ Department of Radiology Binzhou Medical University Hospital Binzhou China; ^4^ College of Basic Medicine Binzhou Medical University Yantai China

**Keywords:** engeletin, HMGB1, inflammation, ischemic stroke, NF‐κB, TLR4

## Abstract

High‐mobility group box1 (HMGB1) induces inflammatory injury, and emerging reports suggest that it is critical for brain ischemia reperfusion. Engeletin, a natural *Smilax glabra rhizomilax* derivative, is reported to possess anti‐inflammatory activity. Herein, we examined the mechanism of engeletin‐mediated neuroprotection in rats having transient middle cerebral artery occlusion (tMCAO) against cerebral ischemia reperfusion injury. Male SD rats were induced using a 1.5 h tMCAO, following by reperfusion for 22.5 h. Engeletin (15, 30 or 60 mg/kg) was intravenously administered immediately following 0.5 h of ischemia. Based on our results, engeletin, in a dose‐dependent fashion, reduced neurological deficits, infarct size, histopathological alterations, brain edema and inflammatory factors, namely, circulating IL‐1β, TNF‐α, IL‐6 and IFN‐γ. Furthermore, engeletin treatment markedly reduced neuronal apoptosis, which, in turn, elevated Bcl‐2 protein levels, while suppressing Bax and Cleaved Caspase‐3 protein levels. Meanwhile, engeletin significantly reduces overall expressions of HMGB1, TLR4, and NF‐κB and attenuated nuclear transfer of nuclear factor kappa B (NF‐κB) p65 in ischemic cortical tissue. In conclusion, engeletin strongly prevents focal cerebral ischemia via suppression of the HMGB1/TLR4/NF‐κB inflammatory network.

## 1 INTRODUCTION

Ischemic stroke (IS) is among the largest contributors to patient mortality and disability around the world.^1^ It is typically caused by increased cholesterol, diabetes mellitus, transient ischemic attack, elevated blood pressure and atrial fibrillation. Following IS, the only globally approved treatment strategy is to ensure rapid blood flow restoration and reoxygenation, and minimize the probability of permanent damage.^2^, ^3^ Unfortunately, increasing reports suggest that reperfusion following IS triggers an inflammatory cascade which results in substantial secondary neuronal damage.^4^

A nuclear nonhistone proinflammatory cytokine called high‐mobility group box 1 (HMGB1) protein is secreted into the extracellular space in response to injury, infection and inflammation.^5^, ^6^ Upregulated circulating HMGB1 content is typically correlated with worse IS patient outcome, and can serve as a precise prognostic indicator following IS.^7^ The HMGB1 from diseased and dying neural cells is released by the IS brain. Significantly, HMGB1 once secreted interacts with myeloid differentiation factor 2, an extracellular adaptor protein that is part of the Toll‐like receptor 4 (TLR4) signalosome, resulting in the TLR4 activation and the subsequent generation of proinflammatory cytokines.^8^, ^9^ TLR4 recognizes and interacts with a wide variety of endogenous and exogenous ligands to control the immune system and defensive mechanisms.^10^ As a result of cerebral ischemia, TLR4 regulates the levels of inflammatory mediators and promotes downstream signalling, including activation of the nuclear factor kappa B (NF‐κB) transcription factor, which then interacts with multiple gene promoters and enhancers to influence a wide range of physiological activities, including inflammation, haematogenesis, immune response, cell proliferation and programmed cell death, which ultimately induces neuroinflammatory responses and injury.^11^, ^12^, ^13^ As a result, a therapeutic approach based on the HMGB1/TLR4/NF‐κB axis can potentially minimize cerebral stroke damage by abrogating neuroinflammation (^14^, ^15^). Thus, the advancement of anti‐inflammatory drugs is very advantageous for treating brain damage brought on by IS.

**FIGURE 1:**
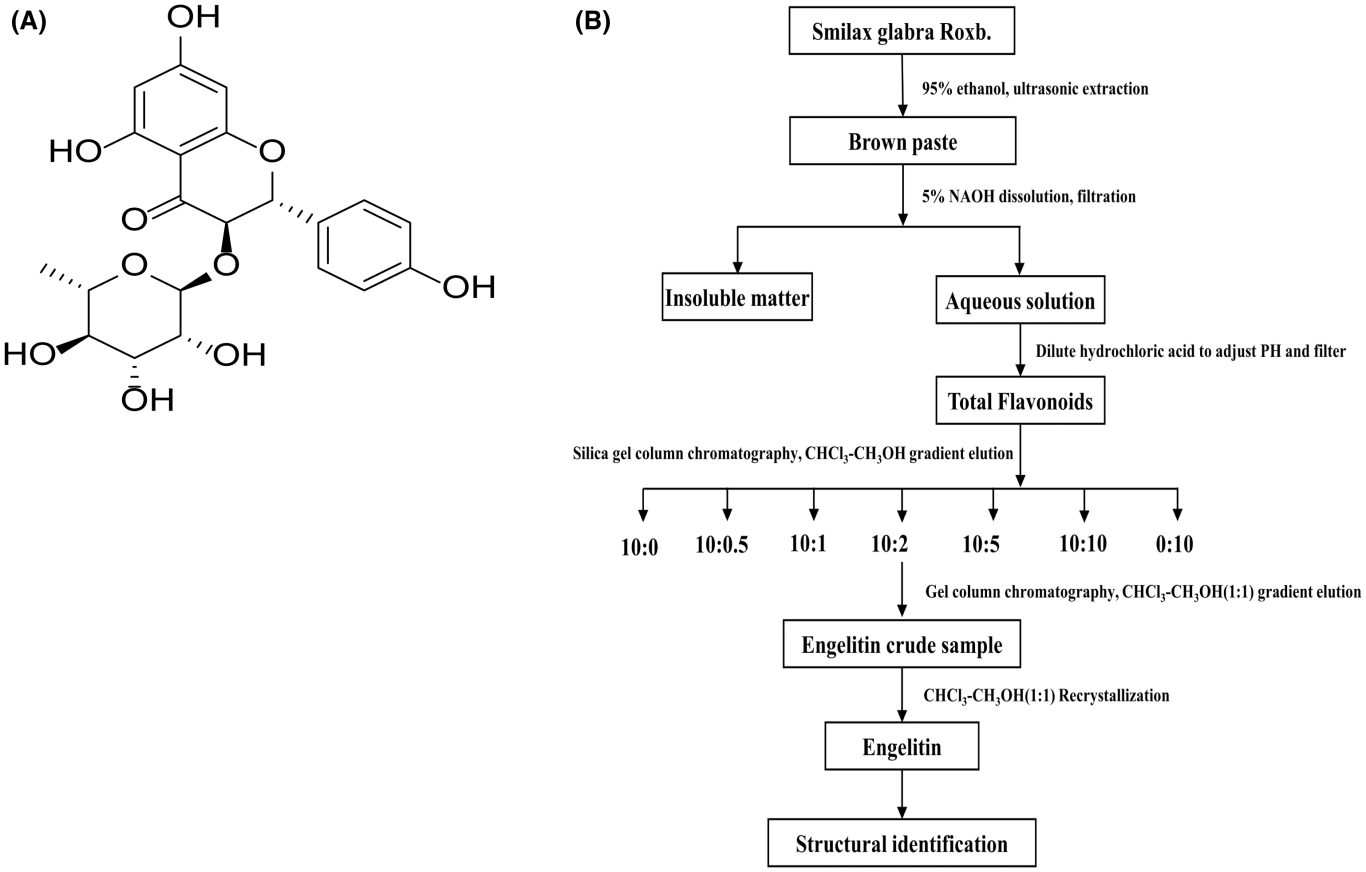
Engeletin structure and isolation. (A) Chemical structure of engeletin. (B) Technical route of separation and extraction of engeletin.

Engeletin is a dihydroflavonoid glycoside compound extracted from a *Smilax glabra rhizomilax derivative*. The chemical structural formula of engeletin is shown in Figure 1A. In a preliminary examination, we determined the engeletin content in Vietnam *Smilax glabra rhizomilax* to be 0.0575%, using the one‐point external standard method, as shown in Figure 1B.^16^ In addition, our team had applied for a patent for invention: The application of engeletin in the preparation of drugs for treating or preventing diabetes induced stroke. We found that the oral dosage range of engeletin for diabetes stroke is 25–1000 mg, preferably 25–500 mg. Emerging reports suggest strong neuroprotective properties of engeletin against multiple diseases, namely, brain stroke, pulmonary fibrosis and Alzheimer's disease.^17^, ^18^ Given that inflammatory stress contributes to IS‐induced brain injury, and that engeletin is reported as a robust anti‐inflammatory agent, it is possible that engeletin may be novel therapeutic approach for cerebral ischemia pretreatment. With this in mind, herein, we assessed the engeletin‐mediated neuroprotection against IS, and illustrated a potential mechanism involving the HMGB1/TLR4/NF‐κB network of signal transduction.

## 2 MATERIAL AND METHODS

### 2.1 Drugs and reagents

We resuspended engeletin (>99.0% pure, MW = 434.39) in dimethyl sulfoxide (DMSO). For dose–response studies, rats were randomly assigned to six different treatment groups: sham group, tMCAO group, edaravone group (6 mg/kg) and engeletin groups (15, 30 and 60 mg/kg). Antibodies (HMGB1: ab79823, TLR4: ab22048, Bcl‐2: ab32124, NF‐κB p65: ab16502, Bax: ab182733 and Cleaved Caspase‐3: ab214430) were acquired via Abcam (Cambridge, MA, United States), and anti‐GAPDH (AF2819) from Beyotime Biotechnology (Shanghai, China).

### 2.2 Animals and middle cerebral artery occlusion surgery

The male Sprague Dawley rats were purchased from Jinan Pengyue Experimental Animal Breeding Co., Ltd. (Certificate No. SYXK 2017 0018, SPF grade) weighed between 220 and 250 g. The tMCAO surgery was conducted on mice via a intraluminal suturing technique first reported by Longa et al. All rats received anaesthesia via pentobarbital (50 mg/kg administered via intraperitoneal route). To properly occlude the middle cerebral artery (MCA), the internal carotid artery (ICA), the external carotid artery (ECA) and the right common carotid artery (CCA), were gently separated, and the CCA was then incised. After that, an 18–20 mm long and 0.285 mm diameter polylysine coated monofilament nylon suture was threaded through the ICA. After the 1.5 h procedure, the nylon suture was removed MCA and reperfusion was enabled to occur. The identical surgical blood vascular separation was performed on rats that had been sham‐operated, but no occlusion was applied. The rats were maintained at 37°C throughout the investigation process.

### 2.3 Neurological deficit score estimation

After 24 h post tMCAO, we assessed the neurological deficit scores based on the Longa et al. reported procedure.^19^ Score 0 indicated lack of neurological deficit; 1 indicated left forepaw extension failure; 2 represented left circling of the paw; 3 represented failure to turn left; and 4 represented spontaneous mobility failure and a depressed consciousness. An elevated neurological deficit score represented more severe motor injury.

### 2.4 Determination of infarct volume

Following rat sacrifice, the extracted brains were sliced into five coronal slices of 2 mm thickness each. Upon a 30 min staining using 2,3,5‐Triphenyltetrazolium chloride (TTC, 2%, T8170, Solarbio, Beijing, China) without light at 37°C, images were captured using a digital camera, and infarct volume calculation was performed via Image J.

### 2.5 Measurements of brain edema

Brain tissue was weighed immediately (wet weight) and after dehydration (dry weight) at 100°C for 24 h. Brain edema was determined using the following formula:

$\text{Brain water content}=\left(\left[\mathrm{wet}\;\text{weight}-\mathrm{dry}\;\text{weight}\right]/\mathrm{wet}\;\text{weight}\right)\times 100\%$.

### 2.6 Histomorphological analysis

Brain sections underwent fixing in 4% paraformaldehyde, and embedding in paraffin, prior to staining with HE and TUNEL stainings. Following neutral resin sealing, image capture was performed via an optical microscope (SYZX6061, Nikon, Tokyo, Japan). The histomorphological analysis was evaluated by two examiners blinded to the treatment groups.

### 2.7 Enzyme‐linked immunosorbent assay (ELISA)

Blood from the abdominal aorta of rats was subjected to centrifugation at 3000*g* for a period of 20 min at 4°C to collect serum. Circulating inflammatory cytokines, namely, IL‐1β, TNF‐α, IL‐6 and IFN‐γ were assessed via ELISA. In accordance with the directions of the kit, an enzyme‐labelled apparatus (Multiskan GO, Thermo, Waltham, MA, United States) was used to record the absorbance (OD) at 450 nm.

### 2.8 Real‐time reverse transcription‐quantitative PCR (RT‐qPCR) for mRNA expression

To determine the detect the effect of engeletin on mRNA expression, we selected 60 mg/kg of engeletin for engeletin detection. TRIzol (Carlsbad CA92008, United States) was used for total RNA isolation from the ipsilateral ischemic cerebral cortex tissues, as per kit directions. RNA quantification was done via the ultraviolet visible spectrophotometer (Beckman, CA, United States). The conversion of cDNA was carried out at 25°C for 10 min, 42°C for 15 min and 85°C for 5 min. Lastly, Power SYBR® Green PCR Master Mix real‐time PCR reactions were performed in an ABI 7500 sequence detection system using the following conditions: denaturation at 95°C for 3 min; 40 cycles at 95°C for 15 s, 53°C for 30 s; and 72°C for 15 s, in a ABI 7500 sequence detection system (Applied Biosystems Co., California, CA). RT‐qPCR was used to study whether engeletin plays a role in inflammatory related transcription factors in ischemic cerebral cortex. The employed primer sequences were acquired from Sangon Biotech, Shanghai, China, as summarized in Table 1. Moreover, GAPDH was employed as endogenous control, and the relative mRNA target gene expressions were computed via the 2^−ΔΔCt^ approach.

**TABLE 1:** Primer sequences used for RT‐PCR analysis.

Gene	Forward (5′‐3′)	Reverse(5′‐3′)
HMGB1	GGATGCTTCTGTCAACTTCTC	TTCATAACGAGCCTTGTCAG
TLR4	GTCAGTGTGATTGTGGTATCC	ACCCAGTCCTCATTCTGACTC
IL‐1β	AAATGCCACCTTTTGACAGTG	GAGTGATACTGCCTGCCTGA
IL‐6	GACAAAGCCAGAGTCCTTCAGA	GAGCATTGGAAATTGGGGTAGG
TNF‐α	TGCCTCAGCCTCTTCTCATT	GGGCTTGTCACTCGAGTTTT
IFN‐γ	GTATTGCCAAGTTTGAGGTCAAC	GCTTCCTGAGGCTGGATTC
GAPDH	GCAGTGGCAAAGTGGAGATTG	TGCAGGATGCATTGCTGACA

### 2.9 Western blotting

Total protein and cytosolic and nuclear protein in the ischemic cortex tissue were extracted with the corresponding protein extraction kit (Key GEN Biotech, China) following the manufacturer's protocols, and the protein quantity was assessed with the BCA protein assay kit (Key GEN Biotech, China). Equal protein quantities were separated using sulfate polyacrylamide gel electrophoresis (8%–12%), prior to transfer to polyvinylidene difluoride membranes (Millipore, USA). After that, they were blocked for 2 h in 5% milk before being exposed to HMGB1, TLR4, Bax, Bcl‐2, NF‐κB p65, Cleaved Caspase‐3, GAPDH and Lamin B antibodies for an entire night at 4°C. The blots were thrice rinsed for 10 min each, before 1 h incubation in matched horseradish peroxidase‐conjugated secondary antibody at room temperature 2 h. Following three more washes in TBST buffer, protein visualization was done on X‐ray film employing the Super ECL Plus Detection Reagent (Key GEN Biotech, China). Protein band quantification was done via Image J.

Significantly, during the operation, the ipsilateral ischemic cerebral cortex tissues were obtained, respectively, both were 1 cm^3^ in size. Each sample was divided into two parts. One part was stored in liquid nitrogen and prepared for protein and total RNA, whereas the other part was fixed in 4% buffered formalin and prepared for the histomorphological analysis.

## 3 STATISTICAL ANALYSIS

The data are presented as a mean ± SEM. Multigroup tests were performed using one‐way analysis of variance (anova), with post hoc comparisons made using Bonferroni tests and a 95% confidence interval. For the entire data analysis, Prism 8 software (GraphPad Software, San Diego, CA, USA) was utilized. *p* < 0.05 was deemed as a significant value.

## 4 RESULTS

### 4.1 Engeletin attenuated neurological deficit scores

**FIGURE 2:**
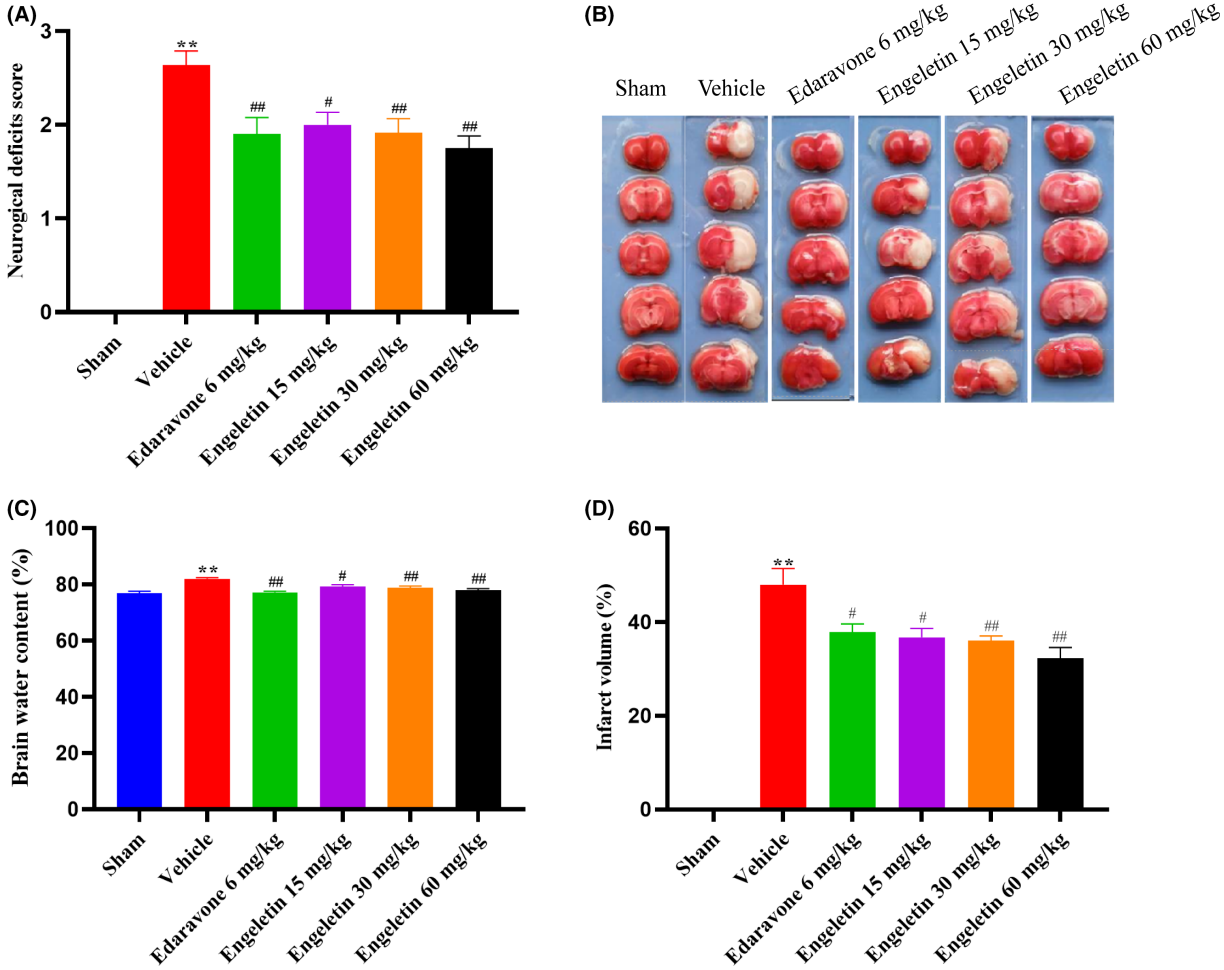
The engeletin‐mediated neuroprotection against ischemic stroke (IS). (A) Evaluation of neurological deficit (*n* = 10–12). (B) Rat brain segments of cerebral ischemia–reperfusion injury stained via TTC in sham, vehicle, edaravone and engeletin (*n* = 5). (C) Brain water content (*n* = 10–12). (D) Infarct volume quantification of sham, vehicle, edaravone and engeletin‐treated samples. One‐way anova was employed for statistical analyses. Mean ± S.E.M. ** *p* < 0.01 vs. sham rats, ^#^ *p* < 0.05, ^##^ *p* < 0.01 vs. vehicle rats.

We examined the engeletin‐mediated protection against ischemia‐induced neurological deficit scores. Although the employed neurological deficit assessment may be variable, it represents the successful formation of the tMCAO model. As depicted in Figure 2A, sham‐operated rats exhibited an neurological deficit score of zero, suggesting no neurological deficit. By contrast, the vehicle‐treated rats displayed a considerably elevated neurological deficit score; however, the engeletin‐treated rats had a substantially reduced neurological deficit, relative to the vehicle‐treated rats (**p < 0.01 vs. sham rats, ^#^ *p* < 0.05, ^##^ *p* < 0.01 vs. vehicle rats; *n* = 10–12 rats/group). As a positive control, we employed edaravone, a newly developed/discovered free radical scavenger that suppresses transient hypoxic–ischemic brain injury. Edaravone exposure also strongly minimized neurological deficit score in tMCAO rats. Likewise, the tMCAO rats with edaravon exposure also displayed considerable neuroprotection, relative to the vehicle‐treated rats (^##^ *p* < 0.01 vs. vehicle rats, *n* = 10 rats/group).

### 4.2 Engeletin diminished infarct volume following focal ischemia

We next assessed how engeletin exposure affected brain infarction employing TTC staining. As illustrated in Figure 2B,D, we administered multiple intravenously injections with varying engeletin (15, 30 and 60 mg/kg) doses following ischemia. Based on our analyses, treatments with varying engeletin doses strongly suppressed the infarct volume, in a dose‐dependent fashion. Among sham‐operated rats, TTC staining of brain slices revealed a uniform red colour, representing healthy tissues. Alternately, the infarct volume from the tMCAO rats displayed a white colour. Relative to the 47.95% ± 7.79% infarct volume in vehicle‐treated rats, the infarct volumes in the engeletin‐treated rats were 36.74% ± 4.26%, 36.01% ± 2.27% and 32.35% ± 7.0%, respectively.

### 4.3 Engeletin drastically reduced the brain water content

Figure 2C shows that vehicle‐treated rats had significantly more brain water than sham‐operated rats. The brain water content was substantially diminished following engeletin, compared to the vehicle‐treated rats (Vehicle vs. 15 mg/kg engeletin rats: 82.05% ± 1.09% vs. 79.30% ± 1.65%, *p* < 0.05; Vehicle vs. 30 mg/kg engeletin‐treated rats: 82.05% ± 3.37% vs. 78.80% ± 1.74%, *p* < 0.01; Vehicle vs. 60 mg/kg engeletin rats: 82.05% ± 3.37% vs. 77.99% ± 1.44%, *p* < 0.01).

### 4.4 Engeletin decreased the degree of brain pathological damage

**FIGURE 3:**
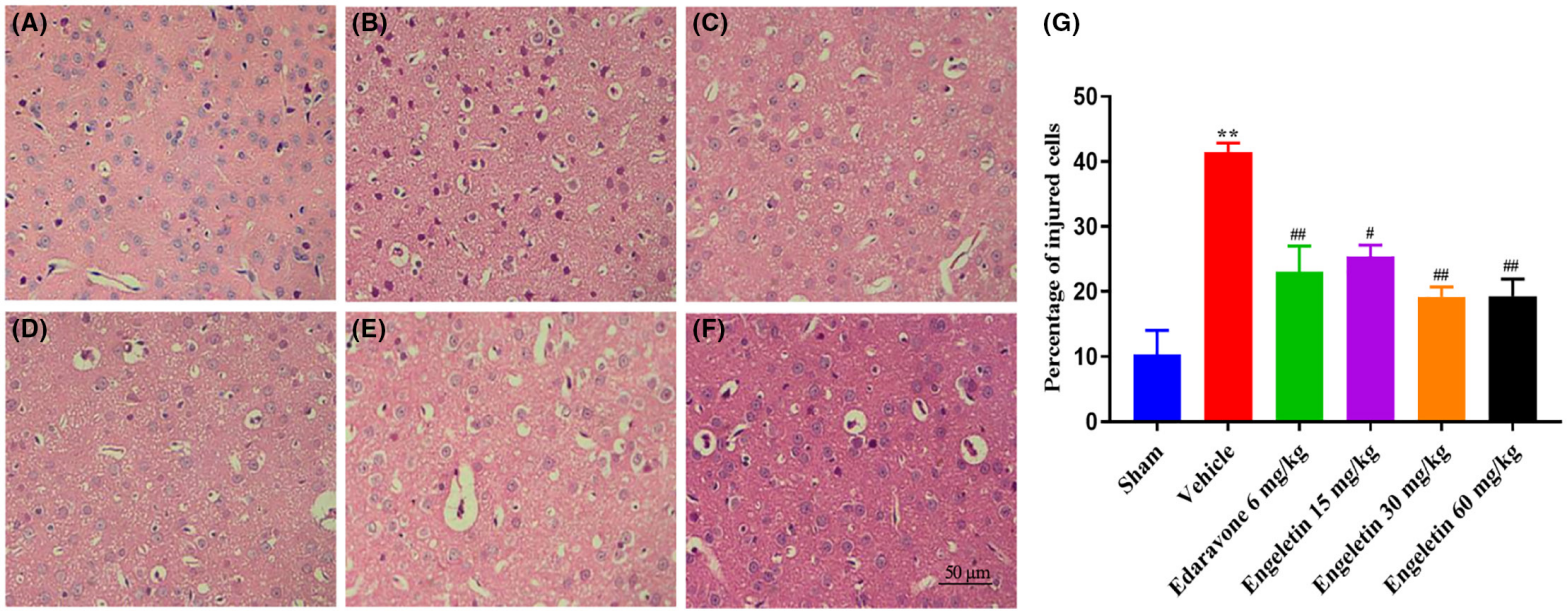
The pathological alterations in the penumbra of rat ischemic ipsilateral parietal cortex were mediated by engeletin at 24 h after tMCAO/R. HE staining images at 400 × magnification. Scale bar = 50 μm. (A) Sham, (B) Vehicle, (C) Edaravone (6 mg/kg), (D) Engeletin (15 mg/kg), (E) Engeletin (30 mg/kg), (F) Engeletin (60 mg/kg). (G) Proportion of injured cells in sham, vehicle, edaravone and engeletin‐treated cerebral cortex samples. Mean ± S.E.M (*n* = 3). **p < 0.01 vs. sham rats, ^#^ *p* < 0.05, ^##^ *p* < 0.01 vs. vehicle rats.

To assess the pathological alterations in the ischemic brain tissue, we performed HE staining (Figure 3). Based on our observation, the positive cells from the sham‐operated rats were intact and abundant, with no immune cell infiltration. The model samples, however, revealed significantly less positive cell nuclei, with an abundance of noticeable disorder among cells, namely, shrunken nucleus, destroyed nucleus and immune cell invasion. By contrast, the engeletin‐treated samples revealed strong amelioration of the aberrant observations found with the untreated tMCAO samples.

### 4.5 Engeletin inhibited neuronal apoptosis caused by tMCAO/R

**FIGURE 4:**
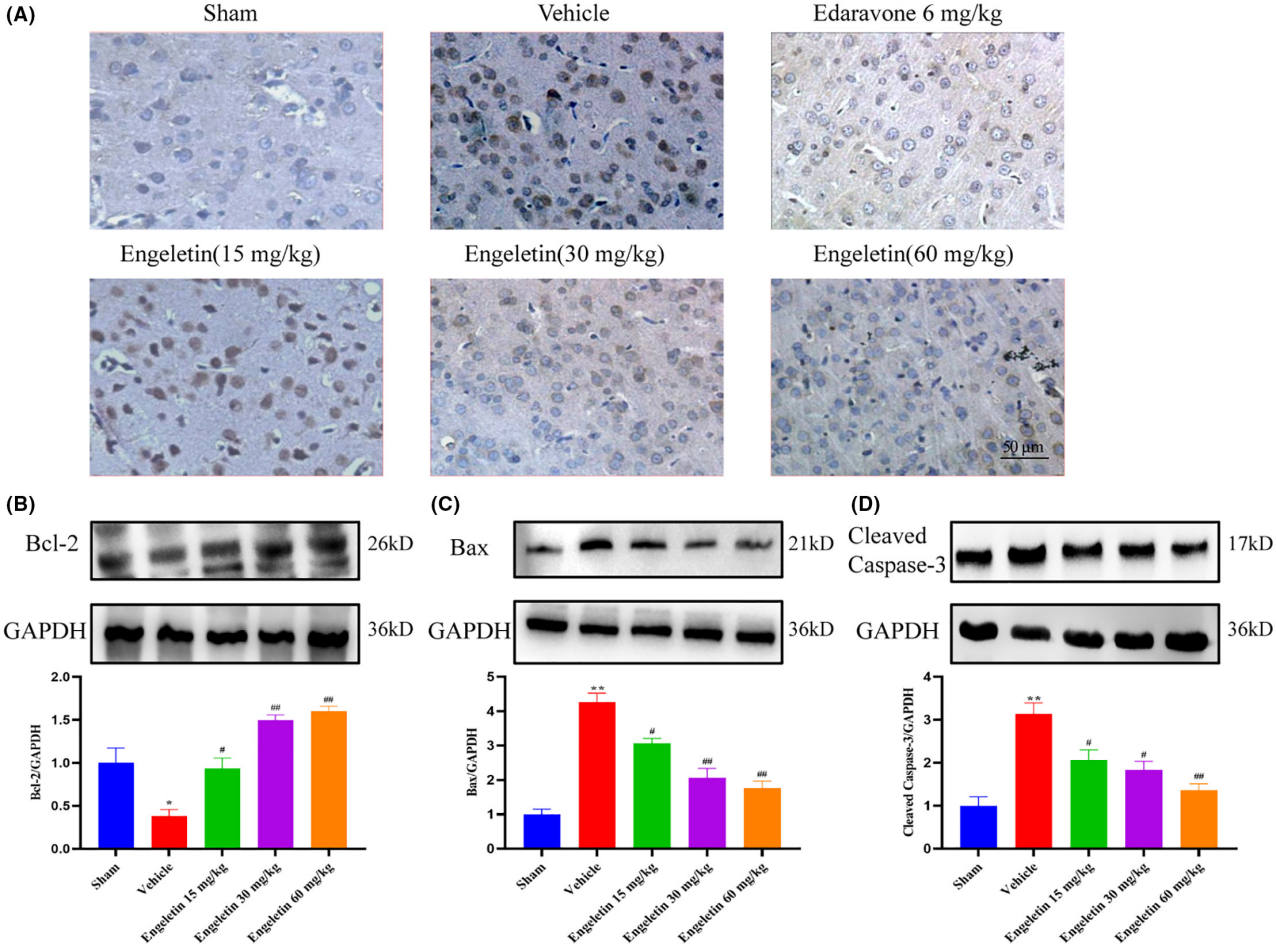
The engeletin‐mediated regulation of apoptotic cortical neuronal death. (A) TUNEL staining images of the penumbra of ischemic ipsilateral parietal cortex region of tMCAO/R rats. TUNEL staining images at 400 × magnification. Scale bar = 50 μm. (B–D) Assessment of rat cortex apoptotic proteins, namely, Bcl‐2, Bax, Cleaved‐Caspase‐3, employing the Western blot analysis. GAPDH was employed as an endogenous reference. Mean ± S.E.M (*n* = 3). **p < 0.01 vs. sham rats, ^#^ *p* < 0.05, ^##^ *p* < 0.01 vs. vehicle rats.

It is well established that apoptosis is a serious pathophysiological event following tMCAO/R injury. Hence, we examined the potential impact of engeletin on neuronal apoptosis using TUNEL staining (Figure 4A). Sham‐operated rats displayed normal neuronal morphology. By contrast, the TUNEL‐positive neurons were enhanced among the vehicle‐treated rats and markedly reduced among the engeletin‐treated rats. Similarly, based on our Western blot analysis, the Cleaved Caspase‐3 and Bax proteins were substantially elevated, whereas, the Bcl‐2 protein was strongly diminished, relative to the sham‐operated rats (Figure 4B–D). Engeletin, however, markedly diminished the Cleaved Caspase‐3 and Bax protein contents, with marked elevations in the Bcl‐2 protein levels (**p < 0.01 vs. sham rats, ^#^ *p* < 0.05, ^##^ *p* < 0.01 vs. vehicle rats; *n* = 3 rat/group).

### 4.6 Engeletin diminished the brain inflammatory cytokine contents

**FIGURE 5:**
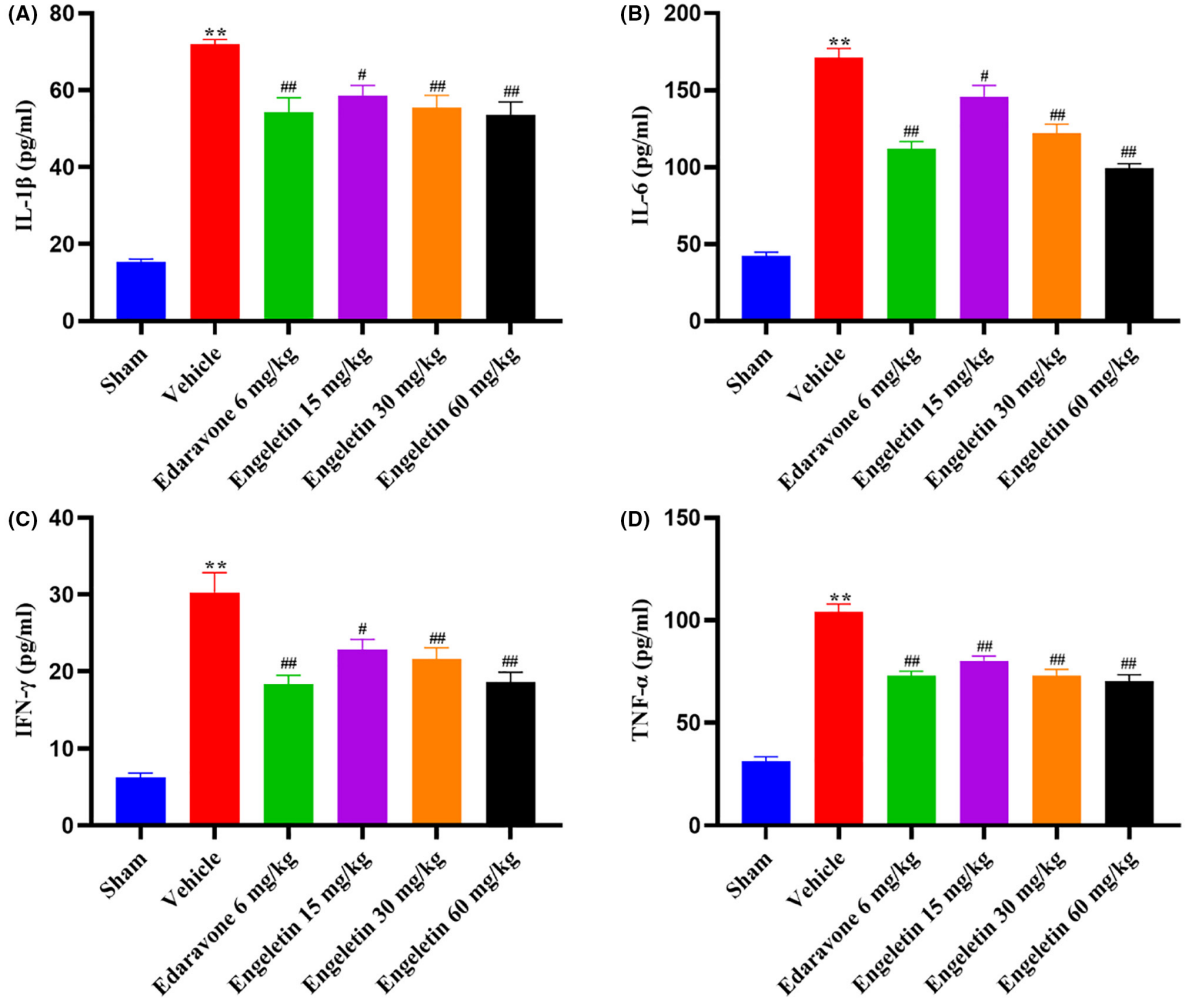
The engeletin‐mediated regulation of circulating inflammatory cytokines in tMCAO/R rats. Serum proinflammatory cytokine expressions, as determined by ELISA kits. (A) IL‐1β, (B) IL‐6, (C) IFN‐γ and (D) TNF‐α. Mean ± S.E.M (*n* = 10–12). **p < 0.01 vs. sham rats, ^#^ *p* < 0.05, ^##^ *p* < 0.01 vs. vehicle rats.

Inflammatory response is critical to IS pathogenesis. Employing ELISA, we measured the quantities of the inflammatory cytokines TNF‐α, IL‐6, IL‐1β and IFN‐γ in this study. As depicted in Figure 5, the aforementioned proinflammatory cytokines were strongly enhanced in vehicle‐treated versus sham‐operated rats. Alternately, engeletin strongly diminished the IL‐6, IL‐1β, TNF‐α and IFN‐γ expressions (**p < 0.01 vs. Sham rats, ^#^ *p* < 0.05, ^##^ *p* < 0.01 vs. Vehicle rats; *n* = 10–12 rats/group).

### 4.7 Engeletin diminished HMGB1, TLR4 and inflammatory cytokine transcript expressions

**FIGURE 6:**
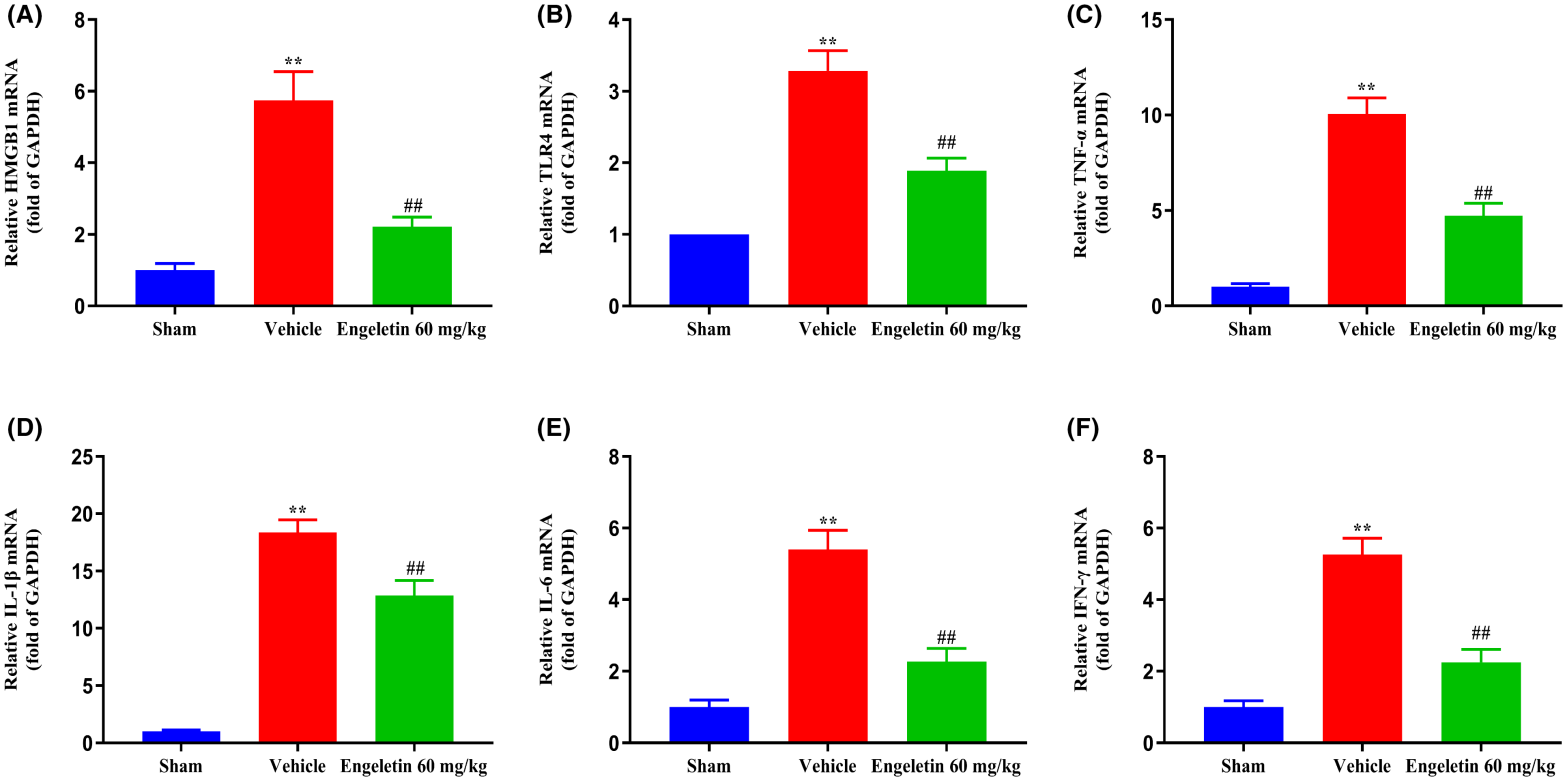
The engeletin‐mediated regulation of HMGB1, TLR4, TNF‐α, IL‐1β, IL‐6 and IFN‐γ transcript expressions in tMCAO/R rats. The relative transcript levels of (A) HMGB1, (B) TLR4, (C) TNF‐α, (D) IL‐1β, (E) IL‐6 and (F) IFN‐γ, as evidenced via quantitative real‐time RT‐PCR. Mean ± S.E.M (*n* = 5). **p < 0.01 vs. sham rats, ^##^ *p* < 0.01 vs. vehicle rats.

As depicted in Figure 6A,B, HMGB1 and TLR4 mRNA expressions were markedly enhanced in vehicle‐treated rats, relative to the sham‐operated rats, and engeletin suppressed their expressions. Lastly, the proinflammatory cytokine TNF‐α, IL‐1β, IL‐6 and IFN‐γ levels were also drastically reduced by engeletin (60 mg/kg) exposure (**p < 0.01 vs. Sham rats, ^##^ *p* < 0.01 vs. Vehicle rats; *n* = 5 rats/group).

### 4.8 Engeletin repressed the HMGB1/TLR4/NF‐κB axis

**FIGURE 7:**
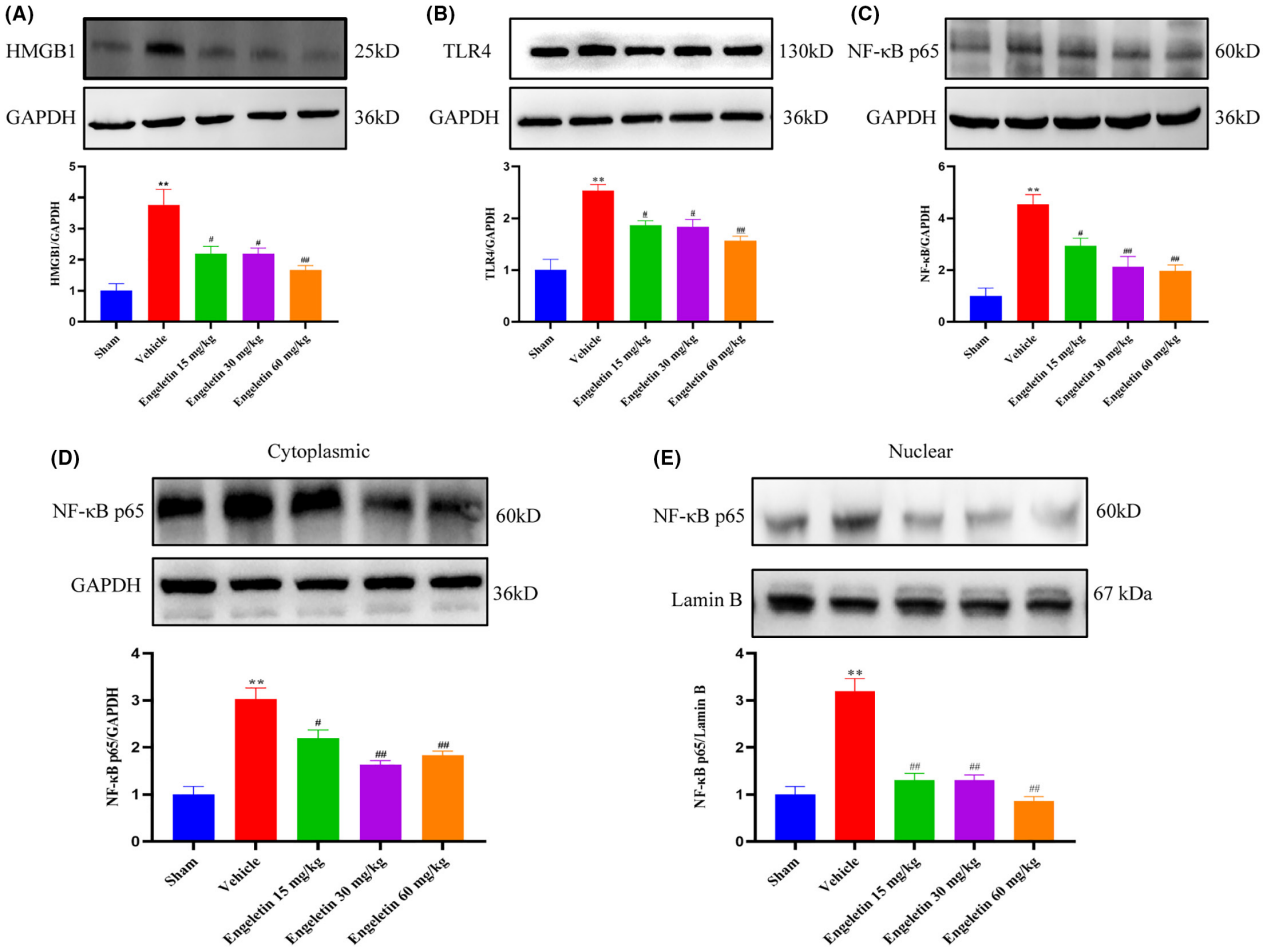
Engeletin downregulated the HMGB1, TLR4, and NF‐κB protein contents and suppressed NF‐κB activation in tMCAO/R rats. (A–C) The HMGB1, TLR4, NF‐κB p65 and GAPDH levels in ischemic brain tissues, as evidenced by Western blot analysis. (D,E) The NF‐κB p65 assessment in cytoplasmic versus nuclear fractions from tMCAO/R rats. Mean ± S.E.M (*n* = 3). **p < 0.01 versus sham rats, ^#^ *p* < 0.05, ^##^ *p* < 0.01 vs. vehicle rats.

Prior investigations revealed that TLR4, HMGB1 and NF‐κB are essential proinflammatory modulators of ischemia‐induced brain injury. Hence, we next examined the engeletin‐mediated regulation of proinflammatory cytokine expression in the ischemic brain following tMCAO/R. To assess the underlying process, we examined the contents of members of the HMGB1/TLR4/NF‐κB axis 24 h post tMCAO/R, using Western blot analysis. As depicted in Figure 7A–C, the NF‐κB p65, TLR4 and HMGB1 protein contents were markedly enhanced following ischemia. The engeletin treatment strongly reduced the ischemia‐induced upregulation of TLR4, HMGB1 and NF‐κB p65 expressions in the ipsilateral hemisphere of the tMCAO/R rats, relative to the vehicle‐treated rats. Generally, NF‐κB p65 remains stable in the cytoplasm under basal conditions. Upon stimulation, NF‐κB p65 translocates to the nucleus to modulate target gene expression. The current findings revealed that the nuclear transport of NF‐κB p65 was substantially increased following ischemia, and it was drastically decreased with engeletin treatment (Figure 7D,E).

## 5 DISCUSSION

Herein, we demonstrated that engeletin treatment drastically enhanced neurological function, and reduced cerebral infarct volume and brain edema following cerebral ischemia reperfusion injury. Moreover, we revealed that engeletin strongly inhibited neuroinflammation via modulation of the TLR4/HMGB1/NF‐κB network. Additionally, the probable pathways underlying engeletin's anti‐inflammatory effects on IS were also investigated. A widely accepted viewpoint is that inflammatory responses are strongly responsible for IS‐induced injury and secondary damage.^20^

Under normal circumstances, HMGB1 stabilizes nucleosomes to regulate gene transcription, and in a number of neurodegenerative disorders, it acts as a late inflammatory cytokine. A prior investigation revealed that circulating HMGB1 contents are generally upregulated by 10‐fold among stroke patients.^21^, ^22^ As such, HMGB1 is a robust stand‐alone indicator of long‐term patient prognosis. It is known that HMGB1 translocation and release trigger the production of proinflammatory cytokines as a result of its interaction with TLR4, which functions as the major HMGB1 receptor.^23^ Moreover, the HMGB1‐TLR4 axis modulates inflammation using several underlying mechanisms.^9^, ^24^ Several evidences suggest that excess inflammation from the brain HMGB1/TLR4 axis activation is strongly contributes to traumatic brain injury and cerebral ischemia reperfusion injury.^25^, ^26^ Hence, suppressing this axis may reduce the expressions of inflammatory genes in cerebral IS.

TLR4 is one axis whereby HMGB1 potentially remains in a feed‐forward loop, leading to the NF‐κB nuclear transfer, a process modulated by both Myd88 and non‐Myd88 axes, to enhance expressions of proinflammatory cytokines.^10^, ^27^ Being a signalling protein, NF‐κB serves an essential function in immunologic and inflammatory response regulation, such as, the p65 and p50 subunit concentrations within the cytoplasm.^28^ Suppressing the TLR4 axis using a targeted inhibitor can prevent neuronal loss, and suppress NF‐κB p65 activation following cardiac arrest and cardiopulmonary resuscitation.^29^, ^30^ Together, these evidences suggest that the HMGB1‐induced postresuscitation‐based brain injury is likely linked to TLR4‐NF‐κB axis.

The transcription factor NF‐kB controls the production of inflammatory cytokines TNF‐α, IL‐1β, IL‐6 and IFN‐γ, as well as growth factors, immunoreceptors and enzymes involved in oxidative stress.^31^, ^32^ A prior investigation revealed that the NF‐κB axis suppression elicits strong neuroprotection, and it minimizes infarct volume, alleviates neurological deficit scores and reduces contents of inflammatory response genes.^33^ Together, these evidences suggest that NF‐κB suppression following acute cerebral infarction may be a robust and efficacious therapy against cerebral ischemia reperfusion injury.

Numerous evidences, involving experimental and clinical trials, indicated that an acute and prolonged inflammatory response promotes ischemic brain injury (^34^, ^35^). Cytokines, namely, IL‐1β, TNF‐α, IL‐6 and IFN‐γ, rise 24 h post ischemia in a tMCAO model, and reducing the concentrations of these cytokines are highly advantageous to IS treatment. Similarly, herein, we revealed that the IL‐1β, TNF‐α, IL‐6 and IFN‐γ mRNA contents enhanced after the development of ischemia in tMCAO stroke rats, whereas, these expressions were diminished after engeletin treatment. This indicated that engeletin abrogated inflammatory responses both during and after acute ischemic brain injury. Additionally, this inhibition contributed to enhanced brain function.

Engeletin is a frequently studied natural compound with numerous physiological activities. Its advantages in treating and preventing disease have been confirmed in multiple reports. In particular, engeletin was shown to diminish cerebral ischemia reperfusion injury by regulating the vasohibin and Ang‐1/VEGF/Tie‐2 axes.^16^ Herein, our findings corroborated with others in that engeletin demonstrated a neuroprotective nature against cerebral ischemia reperfusion injury. We also presented a novel underlying mechanism involving the HMGB1/TLR4/NF‐κB axis. We revealed that engeletin strongly abrogated the nuclear transfer of NF‐κB, which not only relieved neurological deficit scores, but also minimized brain edema, infarct volume and affected cortical area following IS, as was evidenced by HE and TUNEL stainings in vivo.

**FIGURE 8:**
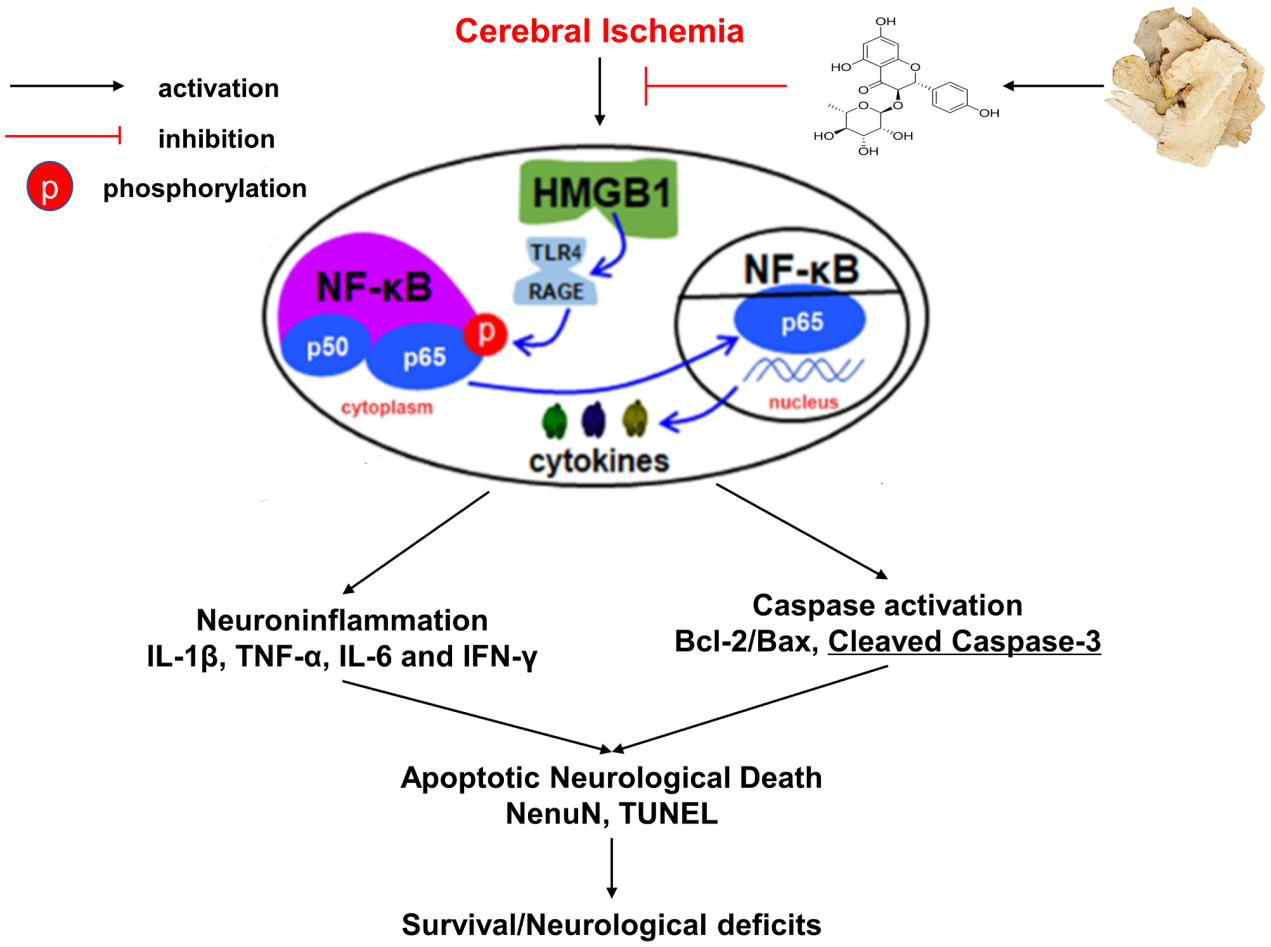
An illustration of the potential engeletin‐based mechanism of neuroprotection in tMCAO/R rats using the HMGB1/TLR4/NF‐κB axis. Cerebral ischemia induces the HMGB1/TLR4 pathway, along with IκBα phosphorylation, thereby accelerating the nuclear transfer of NF‐κB from the cytoplasm. This, in turn, activates target gene expression and the production of numerous cytokines, which enhance neuroinflammation and caspase activation, thereby inducing neuronal apoptosis.

In conclusion, as shown in Figure 8, our experimental results revealed that engeletin strongly protects against neuronal damage during cerebral ischemia reperfusion injury, likely via reduction in HMGB1 release and the NF‐κB nuclear transfer, which may be a direct or indirect cause of neuroinflammation suppression. Hence, we speculate that the engeletin‐mediated regulation of associations among HMGB1, TLR4 and NF‐κB are potentially beneficial to protecting against IS, as was evidenced by the delayed damage following acute and massive neuroinflammation. Of course, further investigations including toxicity, IC50, extinction dose, possible active metabolites brain barrier penetration are needed to further elucidate the mechanisms of engeletin before its application in patients affected by cerebral ischemic attack.

## AUTHOR CONTRIBUTIONS

**Yangyang Xu:** Data curation (lead); funding acquisition (lead); methodology (equal); project administration (lead). **Jie Zhang:** Data curation (supporting); formal analysis (lead); funding acquisition (equal). **Fei Gao:** Methodology (supporting). **Wenna Cheng:** Data curation (supporting); methodology (supporting). **Ye Zhang:** Formal analysis (supporting); methodology (supporting). **Chuanmei Wei:** Conceptualization (supporting); formal analysis (supporting); project administration (supporting). **Shuping Zhang:** Conceptualization (lead); data curation (supporting); funding acquisition (supporting). **Xin fu Gao:** Conceptualization (lead); funding acquisition (lead); project administration (supporting).

## CONFLICT OF INTEREST STATEMENT

The authors declare no competing financial interests.

## Data Availability

The original contributions presented in the study are included in the article/Supplementary Material, further inquiries can be directed to the corresponding authors.
